# Global, regional, and national burden of liver cancer attributable to high BMI, 1990-2021, with future projections to 2040

**DOI:** 10.3389/fonc.2025.1630875

**Published:** 2026-01-27

**Authors:** Teng Ma, Yawen Lu, Kaiyan Liu, Na Liu, Qian Liu, Ziyuan Ding, Jianfeng Ma, Yongfeng Wang, Sheng Li

**Affiliations:** 1 The Second Clinical Medical College of Lanzhou University, Lanzhou, Gansu, China; 2 The First Clinical Medical College of Lanzhou University, Lanzhou, Gansu, China; 3 School of stomatology, Lanzhou University, Lanzhou, Gansu, China; 4 School of Public Health Lanzhou University, Lanzhou, Gansu, China; 5 Key Laboratory of Molecular Diagnostics and Precision Medicine for Surgical Oncology in Gansu Province, Gansu Provincial Hospital, Gansu, China; 6 NHC Key Laboratory of Diagnosis and Therapy of Gastrointestinal Tumor, Gansu Provincial Hospital, Lanzhou, China; 7 The First Hospital of Lanzhou University, Lanzhou, Gansu, China

**Keywords:** age-period-cohort model, global burden of disease, high body-mass index, joinpoint regression analysis, liver cancer

## Abstract

**Background:**

High body mass index (BMI) is a key metabolic risk for liver cancer, yet global assessments of its impact are limited. This study analyzes the liver cancer burden linked to high BMI from 1990 to 2021, using frontier analysis to evaluate prevention efficiency at different SDI levels and providing future predictions of the global burden up to 2040 to guide public health strategies.

**Method:**

We analyzed global burden mortality and disability rates using 2021 data, applying age-period-cohort modeling to assess the temporal dynamics of BMI-related liver cancer burden, adjusting for period and cohort effects across 204 countries and 21 regions, stratified by sex, age, and Sociodemographic Index (SDI). The analysis incorporated frontier efficiency estimates to understand the best achievable burden reduction at each SDI level and utilized a Bayesian framework for future trend predictions.

**Result:**

From 1990 to 2021, high BMI-related liver cancer deaths reached 46,200.88 worldwide, predominantly affecting men, especially ages 70–74 and 60–64 for DALYs. The disease burden varies nonlinearly with SDI, with regional disparities: Southeastern South America and Central/Eastern Europe face the highest impact, despite higher SDIs in Japan, South Korea, and Singapore, where the burden is comparatively lower. Frontier analysis reveals that countries with higher SDI levels have optimized prevention and control strategies, achieving lower burdens, while lower SDI regions still face significant challenges. The future projections indicate that the global burden of high BMI-related liver cancer will continue to rise by 2040, with rapid increases in male populations, although female predictions show more gradual growth.

**Conclusion:**

High BMI contributes to the increasing and unequal global burden of liver cancer, mainly impacting men, seniors, and middle-to-high SDI areas. Urgent, targeted efforts are needed to combat obesity, metabolic issues, and healthcare inequalities to reduce future risks. Future prevention strategies should incorporate continuous monitoring and adaptive responses to dynamic factors influencing liver cancer attributable to high BMI.

## 1 Introduction

Liver cancer, a malignant tumor that takes root in the liver tissue (1–3), is a major player in cancer-related fatalities across the globe. The International Agency for Research on Cancer pegged it as the sixth most prevalent cancer worldwide in 2020. Sadly, it also ranks third as a cause of cancer deaths. It is roughly 906,000 fresh cases, chipping in about 4.7% to the overall cancer caseload, and a staggering 830,000 deaths, accounting for a hefty 8.3% of all cancer-related deaths. Over the past few years, liver cancer rates have been climbing worldwide, especially in Southeast Asia and Africa. Pinpointing the precise cause is still a work in progress, but research points to a mix of factors. These include chronic viral hepatitis (such as Hepatitis B and C), alcoholic liver disease, non-alcoholic fatty liver disease (NAFLD), genetic predisposition, and environmental factors (3–7). Obesity, type 2 diabetes and NAFLD are replacing viral and alcoholic liver disease as the leading causes of disease. Since June 2023, Metabolic Dysfunction-Associated Steatotic Liver Disease (MASLD) has served as the updated term for NAFLD, which is the most prevalent liver disease worldwide and a leading cause of morbidity and mortality related to liver-associated conditions (8). This paper focuses on the global burden of liver cancer caused by obesity.

The epidemic of obesity has reached epidemic proportions and has recently increased dramatically. Body Mass Index (BMI), calculated as weight in kilograms divided by height in meters squared (kg/m^2^), serves as a key metric for evaluating obesity. The World Health Organization (WHO) classifies a BMI of 25 kg/m^2^ or higher as overweight and 30 kg/m^2^ or above as obese. Research (9) has consistently linked elevated BMI to the onset and progression of liver cancer, specifically hepatocellular carcinoma (HCC). A large-scale U.S. study by Campbell et al. (10) tracking 1.57 million adults demonstrated a clear correlation between higher BMI and increased HCC risk, highlighting BMI’s potential as an early warning sign. Additionally, Sohn et al.’s (11) longitudinal study reinforced these findings, showing that obese individuals face not only greater susceptibility to HCC but also higher mortality rates—solidifying BMI as an independent predictor of both disease development and poor outcomes.

Liver cancer incidence is rising, partly due to rising BMI. These metabolic factors may become significant risks in Western countries in the next few years (12). While elevated BMI is widely acknowledged as a major contributor to liver cancer risk, comprehensive global analyses of its disease burden among adults aged 20+ remain scarce. Current research (13–15) tends to focus narrowly on particular nations or localities, offering limited insight into how this burden varies across socioeconomic contexts, age brackets, and gender. What’s more, few studies have examined the longitudinal patterns of BMI-related liver cancer at the population level—a critical oversight given the relentless climb in worldwide obesity prevalence (16) and its uncertain implications for cancer prevention efforts. This study seeks to bridge these gaps by conducting a thorough evaluation of BMI-attributable liver cancer burden from 1990 through 2021, mapping its geographic and temporal patterns to inform more precise, data-driven public health strategies.

This study is based on the 2021 Global Burden of Disease (GBD 2021) dataset, examining trends in liver cancer associated with high BMI from 1990 to 2021, and projected the indicators for 2040. Using methods such as age-period-quota models, Joinpoint regression analysis, and health inequality analysis, this study evaluates changes in age, sex, GBD subcontinental region and country, and social demographic index (SDI) quintiles, providing critical information for formulating policies and interventions to reduce global obesity rates, prevent liver cancer, and protect high-risk populations.

## 2 Materials and methods

### 2.1 Data source and definitions

For this study, we mined data from the GBD 2021 database (https://ghdx.healthdata.org/gbd-results-tool). This comprehensive resource offers a wealth of information, including prevalence, incidence, mortality rates, and overall disease burden, broken down by region, gender, age group, and time period. The data itself is drawn from a variety of disease surveillance, registration, and reporting networks (17). Specifically, we pulled data related to liver cancer on a global scale, spanning the years 1990 to 2021. This included information on prevalence and incidence rates, mortality figures, DALYs, years lived with disability (YLDs) and years of life lost (YLLs), all analyzed by gender and age. Liver cancer cases are classified using the International Classification of Diseases Tenth Edition (ICD-10) standard, identified by the code C22, which encompasses malignant neoplasms of the liver and intrahepatic bile ducts. This classification includes HCC, cholangiocarcinoma, and other primary liver cancer subtypes. High BMI was defined as BMI≥25 kg/m2 for adults (aged ≥ 20 years) and using thresholds from the International Obesity Task Force standards for children (aged< 20 years). This study strictly adheres to the best reporting standards established by the Guidelines for Accurate and Transparent Health Estimates Reporting (GATHER) (18).

### 2.2 Basic study variables

This study’s key variables encompass both exposure factors and metrics of disease burden. On the exposure side, we zeroed in on elevated BMI (BMI ≥ 25 kg/m²), pulling data from population health surveys across different countries and leveraging published meta-analyses. When it comes to disease burden, we looked at the incidence, mortality, DALYs, YLDs, and YLLs specifically related to liver cancer. To keep things consistent and account for differences in age, we age-standardized all rates using direct standardization, referencing the GBD 2021 standard population. In essence, everything was age-adjusted for a fair comparison. To keep tabs on how age-standardized incidence shifts over time, researchers rely on the estimated annual percentage change (EAPC) as a key metric. Mortality stats, disability-adjusted life years, and both ASMR and ASDR figures are all presented with their corresponding 95% uncertainty intervals (19). Regression equations are employed to model the age-standardized rate (ASR), using the year as the predictor variable (x).

The study involved a multi-layered analysis of the data, breaking it down by SDI, gender, age brackets (starting at 20, then grouped in five-year increments), and the 21 geographic areas defined by the GBD study. The SDI, a comprehensive yardstick for gauging developmental progress, scores regions from 0 to 1. It’s figured out by averaging three things: how much people earn, education levels of adults (15+), and fertility rates in younger female (under 25). Because a region’s financial state has a big impact on public health, the SDI is used to evaluate developmental progress throughout the GBD regions. Globally, these regions are sorted into five SDI categories, spanning from low to high. A 95% uncertainty interval (UI) is included to show how reliable the data is.

### 2.3 Study analysis

All statistical analyses were performed using R (version 4.1.2). Significant bilateral *p* values were defined as less than 0.05.

#### 2.3.1 Preliminary analysis

This study analyzed global epidemiological trends for liver cancer across 204 countries and territories, examining key indicators such as death rates, incidence figures, DALY, YLL, and YLD. To account for variations in population demographics, the dataset underwent rigorous standardization employing advanced statistical techniques—including spatiotemporal Gaussian process regression and DisMod-MR meta-regression. This methodological approach yielded refined metrics: ASMR and ASDR, providing comparable insights across diverse populations. To assess the evolving burden of liver cancer, we employed the EAPC as a metric to track shifts in age-standardized incidence rates across a specified timeframe. By defining x as the year, the EAPC was derived using the formula 100 × (exp(β)-1), where β represents the slope from the linear regression model ln(ASR) =α+β· x+ϵ. Consequently, based on this model and its 95% confidence interval (20), an increasing ASR is inferred if the lower bound of the 95% confidence interval (CI) for the predicted EAPC sits above zero. Conversely, a decreasing trend in ASR is signaled if the upper bound of the EAPC’s 95% confidence interval dips below zero.

#### 2.3.2 Age-period-cohort model

An essential statistical technique for resolving trends in time series data and differentiating the effects of cohort, age, and period on the outcome variables is the Age-Period-Cohort model. Building a multivariate regression model is the fundamental idea behind the APC model:


$$Y_{t}=\beta_{0}+\beta_{1}\;Age_{t}+\beta_{2}\;Period_{t}+\beta_{3}\;Cohort_{t}+\epsilon_{t}$$

$Y_{t}$ represents the outcome variables on the $\;Age_{t}$, $\;Period_{t}$ or $Cohort_{t}$. $\;Age_{t}$, $\;Period_{t}$ or $Cohort_{t}$ represents the outcome variables on the *β*_1_, *β*_2_ and *β*_3_. These lines quantified the independent effect of each factor on the outcome variable. $\beta_{0}$ is an intercept term representing the expected value of the outcome variable where all independent variables are zero. $\epsilon_{t}$ is the error term, reflecting the variation that is not explained by the model.

Age-period-cohort analyses were used to disentangle the independent contributions of age, period, and birth cohort to high BMI-attributable liver cancer burden—encompassing deaths, DALYs, YLLs, and YLDs. Age effects were evaluated using longitudinal age curves, which quantified age-specific burden rates (per 100,000 population) across 5-year age groups (0-95+years) stratified by sex. Ninety-five percent UIs were derived from 1,000 posterior draws to account for statistical uncertainty. Period effects were assessed using period risk ratios (RRs), defined as the ratio of disease burden in the high BMI group (≥ 25 kg/m²) to that in the reference group (normal BMI: 18.5-24.9 kg/m²) across calendar periods. These RRs were adjusted for age and other potential confounding risk factors using multivariable mixed-effects models, with 95% UIs estimated via Monte Carlo simulation. Cohort effects were analyzed using cohort RRs, which tracked the association between high BMI and disease burden across 5-year birth cohorts (1900-2000) as these cohorts aged. Cohort RRs adjusted for period effects using APC models to isolate generational differences, with 95% UIs derived from posterior draws of the APC model. All analyses were stratified by sex to explore sex-specific patterns, which was consistent with the framework of the Global Burden of Disease Study 2021.

#### 2.3.3 Joinpoint regression analysis

Joinpoint regression analysis is a method for pinpointing exactly when trends shift within time-series data. It operates by repeatedly challenging the null hypothesis of no trend change. This is achieved by incrementally adding breakpoints, up to three turning points in our analysis, one at a time, while assessing at each step whether the revised model provides a better fit to the data. To determine if these breakpoints are legit, we use Monte Carlo permutation tests with a significance level of 0.05. The model then spits out the annual percentage change (APC) for each segment defined by the breakpoints, as well as the average annual percentage change (AAPC) across the entire time span. We’re particularly interested in the AAPC, and its CI is a key figure we look at. The APC tells us what’s happening locally, whether things are going up, down, or staying put, based on whether its CI includes zero. Meanwhile, the AAPC gives us the big picture view of overall trends.

This method takes a deep dive into the unpredictable ways that liver cancer deaths, DALYs, YLLs, and YLDs fluctuate over time in relation to high BMI. It teases out the specific burdens men and female of different ages face. The SDI, a yardstick that combines income, education, and fertility, gauges a country’s progress. Frontier analysis then pinpoints the absolute lowest possible ASMR and ASDR achievable for each SDI level. By mapping out nonlinear boundaries, like the Health Labor Allocation (HLA) that’s up for grabs, we can figure out the “effective gap”, how much more progress a country could be making in healthcare.

#### 2.3.4 Health inequalities analysis

At its heart, health inequality analysis is all about shining a light on how the burden of disease is spread differently across various socioeconomic groups, using hard numbers to tell the story. The Concentration Index, drawing inspiration from the Lorenz curve, measures the gap between the cumulative distribution of health indicators, things like mortality rates and DALY rates, across a population ranked by their SDI, and a line representing perfect equality. It’s a number between -1 and 1: a negative Concentration Index suggests the disease burden is hitting vulnerable groups hardest, while a positive Concentration Index points to the opposite, with wealthier groups bearing the brunt. Then there’s the Slope Index of Inequality (SII), which takes a different tack. It uses regression analysis to put a number on the absolute difference in health indicators between the highest and lowest SDI groups. In essence, it shows how steeply health outcomes change along the socioeconomic ladder. In this study, we’ve harnessed both these complementary tools: the Concentration Index to get a handle on relative inequality and the SII to gauge the absolute difference. By looking at both, we can paint a comprehensive picture of how the burden of liver cancer linked to high BMI is distributed across socioeconomic strata and how this distribution is changing over time. This, in turn, provides solid, evidence-based insights for crafting targeted intervention strategies. The health inequalities analysis was based on data from the Global Burden of Disease Study 2019, as the dataset for 2021 was not yet available at the time of this study. Therefore, all health inequality metrics presented here are derived from the 2019 dataset.

#### 2.3.5 Frontier analysis

Frontier analysis was employed to examine the most efficient prevention and control strategies for high BMI-related liver cancer burdens at varying SDI levels. This analysis utilized a non-parametric Data Envelopment Analysis-BCC model (with variable returns to scale) combined with regression-based frontier fitting. In this framework, SDI, a comprehensive indicator ranging from 0 to 1 that reflects the socioeconomic development level of each country, was considered the input variable. The output variables, representing the burden, included ASMR and ASDR. The frontier line was established as the theoretical lowest possible burden that could be achieved at a given SDI level, representing the most efficient prevention and control scenario where there is no loss of efficiency at that level of development. For data cleaning, extreme outliers outside the P2.5-P97.5 range in country-year datasets were excluded using the interquartile range method. The frontier line was estimated using the free disposal hull method implemented in the R package “frontier,” and the efficiency score for each country-year was computed as the ratio of actual burden to frontier burden. A score of 1 indicates that the country has reached the highest level of prevention and control efficiency.

#### 2.3.6 Future prediction

To forecast the future trajectory of liver cancer burden attributed to high BMI, a Bayesian framework-based prediction model was employed, designed to align with the structure and standards of the GBD database. The model utilized time-series data for core burden indicators from 1990 to 2021 as the dependent variable, while independent variables incorporated a range of dynamic factors, including SDI trends, GBD-estimated high BMI prevalence rates, population demographics, and macroeconomic indicators. Predictions were made at three levels: global, regional, and SDI quintiles. Markov Chain Monte Carlo (MCMC) methods were used to estimate model parameters, allowing for the quantification of uncertainties. An out-of-sample validation set (comprising data from 2016 to 2021) was employed to assess the performance of the model, with the Root Mean Square Error (RMSE) and Mean Absolute Error (MAE) used to evaluate prediction accuracy. The study incorporated three different scenario simulations: the baseline scenario, an enhanced control scenario, and an efficiency optimization scenario. The model was executed using the R package “Stan,” and the resulting point estimates along with 95% confidence intervals for each health burden indicator between 2022 and 2040 under each scenario provided valuable quantitative data to inform long-term prevention and control policy development.

## 3 Results

### 3.1 Global distribution and trends in liver cancer attributable to high BMI from 1990 to 2021

From 1990 to 2021, liver cancer attributable to high BMI became more common globally. In 2021, the number of liver cancer deaths related to a high BMI was 46,200.88 (95% CI: 38,606.14-77983.02), and the years of life lost due to disability was 1,273,312.58 (95% CI: 504,391.1-2101,957.87). Both figures have more than doubled since 1990. The global ASMR per 100,000 for liver cancer associated with a high BMI increased from 0.26 people (95%UI: 0.11-0.42) in 1990 to 0.53 (95% UI: 0.21-0.90) in 2021, reflecting an EAPC of 106.81% (95% CI: 78.02-132.11) over three decades. Similarly, the ASDR per 100,000 people increased from 76.97 (95% UI: 2.84-11.34) to 14.16 (95% CI: 5.77-24.06), with an EAPC of 103.12% (95% CI: 73.48-129.80) (**Tables 1**, **2**).

**Table 1:** Deaths cases and ASMR per 100,00 population of liver cancer attributable to high BMI in 1990 and 2021.

Characteristics	1990		2021		1990-2021
	Deaths.Cases.No.(95% UI)	ASMR per 100,000 No.(95% UI)	Deaths.Cases.No.(95% UI)	ASMR per 100,000 No.(95% UI)	EAPC(%) in ASMR No.(95% CI)
Global	10282.12 (4196.72,16721.85)	0.26 (0.11,0.42)	46203.88 (18606.14,77983.02)	0.53 (0.21,0.90)	106.81 (78.02,132.11)
Central Europe, Eastern Europe,and Central Asia	1710.54 (701.84,2854.79)	0.36 (0.15,0.59)	3509.70 (1422.39,6036.13)	0.54 (0.22,0.93)	51.56 (28.91,68.17)
High-income	3882.54 (1622.50,6659.73)	0.33 (0.14,0.56)	14785.59 (6131.86,24698.04)	0.69 (0.28,1.14)	110.39 (85.63,128.75)
Latin America and Caribbean	509.66 (204.36,898.17)	0.23 (0.09,0.40)	2439.69 (1028.34,4156.92)	0.39 (0.17,0.67)	71.41 (49.99,91.32)
North Africa and Middle East	1046.85 (375.06,1953.67)	0.62 (0.22,1.16)	5146.86 (2192.41,8603.22)	1.12 (0.48,1.88)	81.37 (13.13,161.75)
South Asia	175.06 (71.36,277.81)	0.03 (0.01,0.04)	1992.31 (787.86,3298.25)	0.13 (0.05,0.21)	353.19 (264.29,460.82)
Southeast Asia, East Asia, and Oceania	2221.03 (914.70,3625.23)	0.18 (0.07,0.30)	15326.59 (6147.09,26950.51)	0.53 (0.21,0.93)	193.74 (114.91,285.29)
Sub-Saharan Africa	736.43 (271.06,1289.94)	0.33 (0.12,0.59)	3003.15 (1186.51,5030.54)	0.61 (0.24,1.00)	81.28 (29.97,167.96)
Gender					
Male	5913.45(2479.64,9717.69)	0.31 (0.13,0.52)	28511.99 (11721.81,49277.60)	0.70 (0.29,1.21)	1.23 (0.91,1.55)
Female	4368.66 (1707.64,7078.83)	0.21 (0.08,0.33)	17691.88 (7169.44,29573.18)	0.38 (0.15,0.64)	0.85 (0.54,1.14)
Socio-demographic Index (SDI)					
Low SDI	419.01 (158.12,750.43)	0.18 (0.07,0.32)	1546.92 (574.00,2670.95)	0.28 (0.11,0.49)	62.25 (24.43,125.71)
Low-middle SDI	1262.13 (483.91,2216.40)	0.21 (0.08,0.37)	6284.68 (2719.55,10345.35)	0.43 (0.18,0.71)	107.68 (43.80,181.80)
Middle SDI	2049.09 (851.37,3300.81)	0.19 (0.08,0.31)	12761.81 (5209.28,21861.01)	0.47 (0.19,0.80)	146.30 (98.81,195.76)
High-middle SDI	3117.67 (1275.19,5231.14)	0.31 (0.13,0.52)	11457.48 (4596.09,20232.07)	0.58 (0.23,1.03)	87.06 (55.98,117.72)
High SDI	3418.41 (1443.94,5783.40)	0.31 (0.13,0.53)	14115.94 (5788.27,23372.16)	0.69 (0.28,1.13)	119.73 (97.76,137.51)

ASMR, age-standardized mortality rate; EAPC, Estimated Annual Percentage Change; UI, uncertainty interval; CI, confidence interval.

**Table 2:** DALYs cases and ASDR per 100,00 population of liver cancer attributable to high BMI in 1990 and 2021.

Characteristics	1990		2021		1990-2021
	DALYs.Cases.No.(95% UI)	ASDR per 100,000 No.(95% UI)	DALYs.Cases.No.(95% UI)	ASDR per 100,000 No.(95% UI)	EAPC(%) in ASDR No.(95% CI)
Global	292696.35 (119094.56,475962.67)	6.97 (2.84,11.34)	1237312.88 (504239.11,2101957.87)	14.16 (5.77,24.06)	103.12 (73.48,129.80)
Central Europe, Eastern Europe,and Central Asia	46185.38 (19009.54,77444.18)	9.49 (3.90,15.88)	88524.38 (35934.85,152677.18)	14.01 (5.69,24.19)	47.72 (25.80,64.81)
High-income	97133.29 (40493.77,167335.42)	8.46 (3.52,14.59)	328418.62 (136151.85,541777.48)	16.91 (7.05,27.78)	99.78 (75.42,118.44)
Latin America and Caribbean	14446.30 (5834.97,25418.14)	6.05 (2.43,10.64)	62645.45 (26446.92,106296.76)	9.88 (4.17,16.76)	63.24 (42.83,82.19)
North Africa and Middle East	30548.44 (11100.36,55989.01)	16.37 (5.89,30.39)	146139.61 (62194.85,241542.35)	29.06 (12.36,48.32)	77.54 (13.42,152.27)
South Asia	5618.19 (2245.11,8906.37)	0.83 (0.33,1.32)	60491.67 (23892.06,101138.04)	3.71 (1.46,6.18)	348.56 (264.97,450.91)
Southeast Asia, East Asia, and Oceania	75626.30 (31206.78,123623.36)	5.67 (2.34,9.25)	456954.10 (183136.11,815771.44)	15.65 (6.28,27.87)	175.90 (98.63,267.72)
Sub-Saharan Africa	23138.45 (8549.83,39514.06)	9.40 (3.46,16.33)	94139.04 (36903.43,158433.86)	16.36 (6.46,27.45)	73.97 (27.20,156.68)
Gender					
Male	178613.48 (74640.79,292177.84)	8.75 (3.66,14.34)	803491.37 (330471.19,1391542.21)	19.05 (7.83,32.99)	1.18 (0.83,1.53)
Female	114082.87 (44523.87,185244.62)	5.26 (2.05,8.53)	433821.51 (178280.70,726288.13)	9.52 (3.91,15.91)	0.81 (0.49,1.10)
Socio-demographic Index (SDI)					
Low SDI	13226.66 (4986.33,23708.75)	5.03 (1.89,9.00)	49951.65 (18543.44,87282.79)	8.17 (3.03,14.20)	62.60 (26.26,128.55)
Low-middle SDI	37310.68 (14411.39,64488.59)	5.51 (2.12,9.63)	183184.42 (78582.95,298065.85)	11.68 (5.02,19.05)	112.03 (50.53,184.23)
Middle SDI	65881.76 (27463.21,105339.60)	5.48 (2.28,8.80)	371886.94 (152202.55,642957.01)	13.09 (5.35,22.58)	138.76 (91.61,190.16)
High-middle SDI	87715.44 (35786.93,146688.08)	8.47 (3.46,14.16)	308350.33 (123611.19,549639.09)	16.05 (6.44,28.59)	89.47 (54.22,126.92)
High SDI	88141.44 (37082.53,149100.53)	8.29 (3.49,13.99)	323005.73 (133944.14,530367.82)	17.19 (7.16,28.19)	107.41 (84.66,125.27)

DALYs, disability-adjusted life years; ASDR, age-Standardized DALYs Rate; EAPC, Estimated Annual Percentage Change; UI, uncertainty interval; CI, confidence interval.

The ASMR per 100,000 of liver cancer in male due to high BMI increased from 0.31 (95% UI: 0.13,0.52) in 1990 to 0.70 (95% UI: 0.29,1.21) in 2021, while the ASMR of female increased from 0.21 (95% UI: 0.08,0.33) in 1990 to 0.38 (95% UI: 0.15,0.64) in 2021. The ASDR of female increased from 5.26 (95% UI: 2.05,8.53) in 1990 to 9.52 (95% UI: 3.91,15.91) in 2021; meanwhile, the ASDR of male increased from 8.75 (95% UI: 3.66,14.34) in 1990 to 19.05 (95% UI: 7.83,32.99) in 2021 (**Tables 1**, **2**).

Globally, the ASMR related to liver cancer has slightly increased, showing different trends in low-, middle-, and high-income regions. The global EAPC is 106.81% (95% CI: 78.02%,132.11%), while the EAPC in high-income regions is 110.39% (95% CI: 85.63%,128.75%). Interestingly, the ASDR related to liver cancer also increased globally, with different trends in low-, middle-, and high-income regions. The global EAPC is 103.12% (95% CI: 73.48%,129.80%), while the EAPC in high-income regions is 99.78% (95% CI: 75.42%,118.44%) (**Tables 1**, **2**).

During the period from 1990 to 2021, the global burden of liver cancer disease was as follows: in 1990, the combined mortality rate and male and female mortality rates were 0.19 per 100,000, 0.22 per 100,000 and 0.16 per 100,000, respectively; in 2021, they were 0.59 per 100,000, 0.72 per 100,000 and 0.45 per 100,000, representing increases of 210.53%, 227.27%, and 181.25%, respectively. The combined DALY rate and male and female DALY rates increased from 5.49 per 100,000, 6.65 per 100,000 and 4.31 per 100,000 to 15.68 per 100,000, 20.29 per 100,000 and 11.03 per 100,000, respectively, with increases of 185.61%, 205.11% and 155.92%. The combined YLD rate and male and female YLD rates increased from 0.05 per 100,000, 0.05 per 100,000 and 0.04 per 100,000 to 0.15 per 100,000,0.20 per 100,000, and 0.11 per 100,000, respectively, with increases of 200.00%, 300.00% and 175.00%. The combined YLL rate and male and female YLL rates increased from 5.44 per 100,000,6.60 per 100,000, and 4.27 per 100,000 to 15.53 per 100,000, 20.10 per 100,000 and 10.92 per 100,000, respectively, with increases of 185.48%, 204.55% and 155. Overall, the combined male and female mortality rate, DALY rate, YLD rate and YLL rate showed a significant increase trend (P< 0.05) from 1990 to 2021 (**Table 3**).

**Table 3:** Temporal trends in deaths rate, DALYs rate, YLDs rate, and YLLs rate per 100,000 population for liver cancer attributable to high BMI from 1990 to 2021.

Year	Deaths rate (per 100,000)			DALYs rate (per 100,000)			YLDs rate (per 100,000)			YLLs rate (per 100,000)		
	Both	Male	Female	Both	Male	Female	Both	Male	Female	Both	Male	Female
1990	0.19	0.22	0.16	5.49	6.65	4.31	0.05	0.05	0.04	5.44	6.60	4.27
1991	0.20	0.23	0.17	5.68	6.93	4.41	0.05	0.06	0.04	5.63	6.87	4.37
1992	0.21	0.24	0.17	5.90	7.24	4.55	0.05	0.06	0.04	5.85	7.18	4.51
1993	0.22	0.25	0.18	6.14	7.58	4.67	0.05	0.06	0.04	6.09	7.52	4.63
1994	0.23	0.26	0.19	6.41	7.97	4.84	0.05	0.07	0.04	6.36	7.90	4.79
1995	0.24	0.28	0.19	6.70	8.38	4.99	0.06	0.07	0.04	6.64	8.31	4.95
1996	0.24	0.29	0.20	6.94	8.74	5.11	0.06	0.07	0.05	6.88	8.67	5.07
1997	0.25	0.30	0.20	7.15	9.02	5.24	0.06	0.08	0.05	7.08	8.94	5.20
1998	0.26	0.31	0.21	7.43	9.45	5.38	0.07	0.08	0.05	7.37	9.37	5.33
1999	0.28	0.33	0.22	7.81	9.97	5.61	0.07	0.09	0.05	7.74	9.88	5.56
2000	0.29	0.35	0.23	8.14	10.46	5.78	0.07	0.09	0.05	8.07	10.37	5.73
2001	0.30	0.36	0.23	8.40	10.81	5.95	0.07	0.09	0.05	8.32	10.72	5.89
2002	0.31	0.37	0.24	8.62	11.08	6.13	0.08	0.10	0.06	8.55	10.98	6.08
2003	0.31	0.38	0.25	8.79	11.27	6.28	0.08	0.10	0.06	8.71	11.17	6.22
2004	0.32	0.39	0.25	8.96	11.50	6.38	0.08	0.10	0.06	8.87	11.39	6.32
2005	0.33	0.40	0.26	9.22	11.84	6.56	0.08	0.11	0.06	9.13	11.73	6.50
2006	0.34	0.41	0.27	9.51	12.22	6.76	0.09	0.11	0.06	9.42	12.11	6.69
2007	0.35	0.43	0.28	9.87	12.72	6.98	0.09	0.12	0.07	9.77	12.60	6.92
2008	0.37	0.45	0.29	10.36	13.38	7.30	0.10	0.12	0.07	10.26	13.26	7.23
2009	0.39	0.47	0.30	10.77	13.94	7.57	0.10	0.13	0.07	10.67	13.81	7.49
2010	0.40	0.49	0.31	11.16	14.47	7.81	0.11	0.14	0.08	11.05	14.34	7.73
2011	0.42	0.51	0.32	11.52	14.98	8.04	0.11	0.14	0.08	11.41	14.83	7.96
2012	0.43	0.53	0.33	11.94	15.51	8.33	0.11	0.15	0.08	11.82	15.36	8.25
2013	0.45	0.55	0.34	12.35	16.07	8.60	0.12	0.15	0.08	12.23	15.92	8.52
2014	0.47	0.58	0.36	12.92	16.82	9.00	0.12	0.16	0.09	12.80	16.66	8.91
2015	0.49	0.61	0.38	13.48	17.57	9.36	0.13	0.17	0.09	13.35	17.40	9.27
2016	0.51	0.63	0.39	13.90	18.05	9.72	0.13	0.17	0.09	13.77	17.88	9.63
2017	0.52	0.64	0.40	14.17	18.38	9.93	0.14	0.18	0.10	14.03	18.20	9.83
2018	0.54	0.66	0.41	14.56	18.89	10.20	0.14	0.18	0.10	14.42	18.71	10.10
2019	0.55	0.68	0.42	14.86	19.27	10.42	0.14	0.19	0.10	14.72	19.09	10.31
2020	0.57	0.70	0.43	15.23	19.76	10.67	0.15	0.19	0.11	15.08	19.57	10.57
2021	0.59	0.72	0.45	15.68	20.29	11.03	0.15	0.20	0.11	15.53	20.10	10.92

DALYs, disability-adjusted life years; YLLs, years lived with disability; YLDs, years of life lost.

### 3.2 Global burden analysis

**Figure 1:**
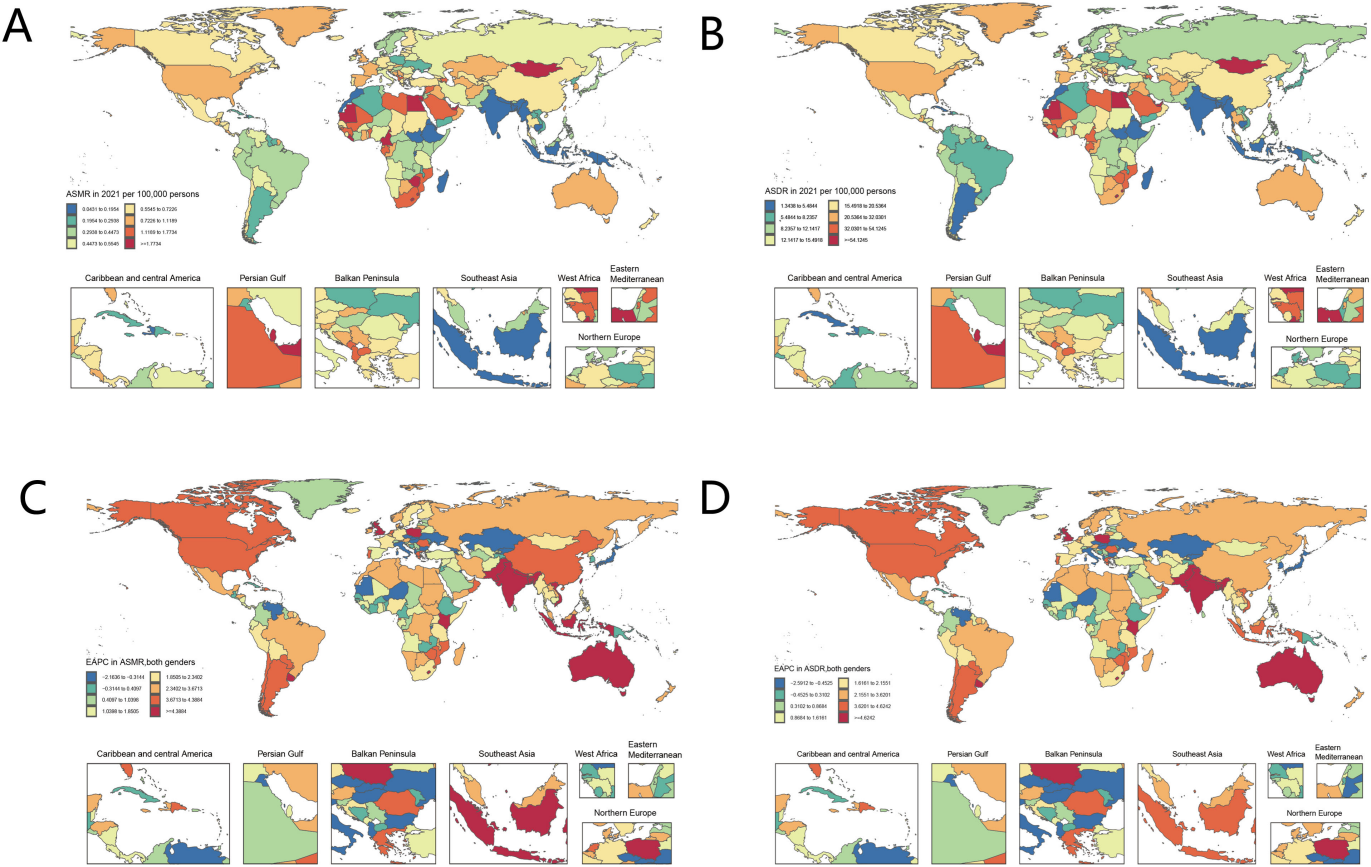
Global distribution of ASR of liver cancer deaths and DALYs attributable to high BMI. ASMR **(A)** and ASDR **(B)** in 2021. The EAPC in ASMR **(C)** and ASDR **(D)**, 1990-2021. DALYs, disability-adjusted life years; ASR, age-standardized rate; ASMR, age-standardized mortality rate; ASDR, age-Standardized DALYs Rate; EAPC, Estimated Annual Percentage Change.

There are significant geographical differences in the spatial distribution of liver cancer burden associated with high BMI in 2021. Mongolia, the Persian Gulf, West Africa, the eastern Mediterranean, and the Balkan Peninsula are the main regions where high ASMR and ASDR were found. On the other hand, the Caribbean and Central America, Southeast Asia, and Northern Europe have lower ASMR and ASDR. The EAPC attributable to high BMI in liver cancer for ASMR and ASDR increased significantly from 1990 to 2021 in North America, Brazil, Argentina, the UK, Poland, Southeast Asia, and Australia, while it decreased significantly in the Balkans, the eastern Mediterranean, most of Africa, Eastern Europe, and northern Central Asia (**Figure 1**).

### 3.3 Age and sex differences and temporal trends in liver cancer attributable to high BMI

**Figure 2:**
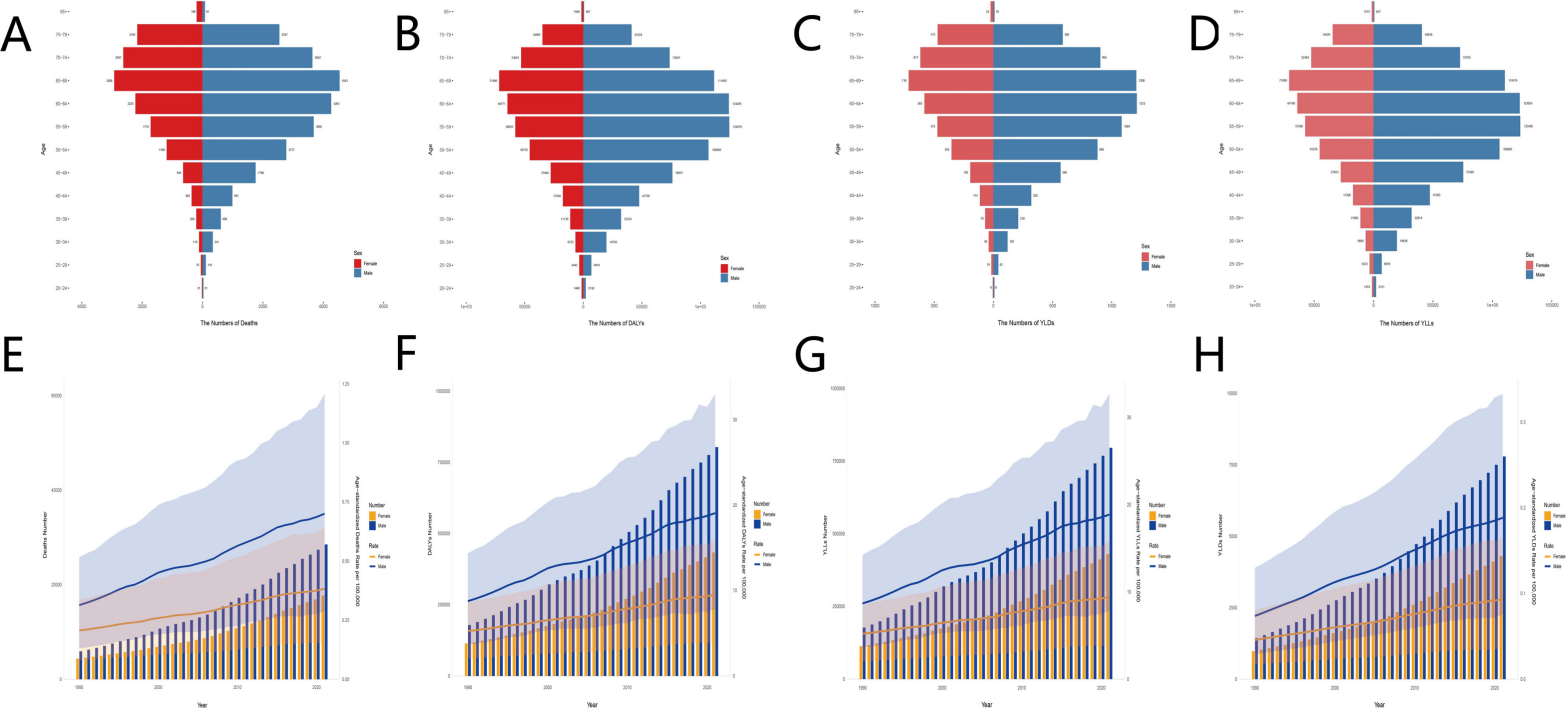
Age- and sex-specific characteristics and temporal trends in the global burden of liver cancer attributable to high BMI. The number of deaths **(A)**, DALYs **(B)**, YLLs **(C)** and YLDs **(D)** for female and male in all age groups. Temporal trends of the number of deaths and ASMR **(E)**, the number of DALYs and ASDR **(F)**, the number of YLLs and ASYLLR **(G)** and the number of YLDs and ASYLDR **(H)**, 1990-2021. DALYs, disability-adjusted life years; ASMR, age-standardized mortality rate; ASDR, age-standardized disability-adjusted life years rate; ASYLLR, Age-Standardized YLLs Rate; ASYDR, Age-Standardized YLDs Rate. Red represents females; blue represents males. The shaded area represents the 95% uncertainty interval.

The global burden of liver cancer attributable to high BMI exhibits unique demographic and temporal patterns. In almost all age groups, the disease burden for males is higher than for females. The number of liver cancer deaths for both males and females increases significantly with age, peaking at ages 65-69 (males: 4,242; females: 2,928). Males have the highest DALYs in the 55–59 and 60–64 age groups (124,579 and 124,265, respectively), while females have the highest DALYs in the 65–69 age group (71,806). The number of YLLs and YLDs follows similar trends to DALYs. Temporal analysis shows that from 1990 to 2021, both males and females exhibit an increasing trend with age, with the disease burden for males consistently higher than for females (**Figure 2**).

### 3.4 Results of the age-period-cohort analysis

**Figure 3:**
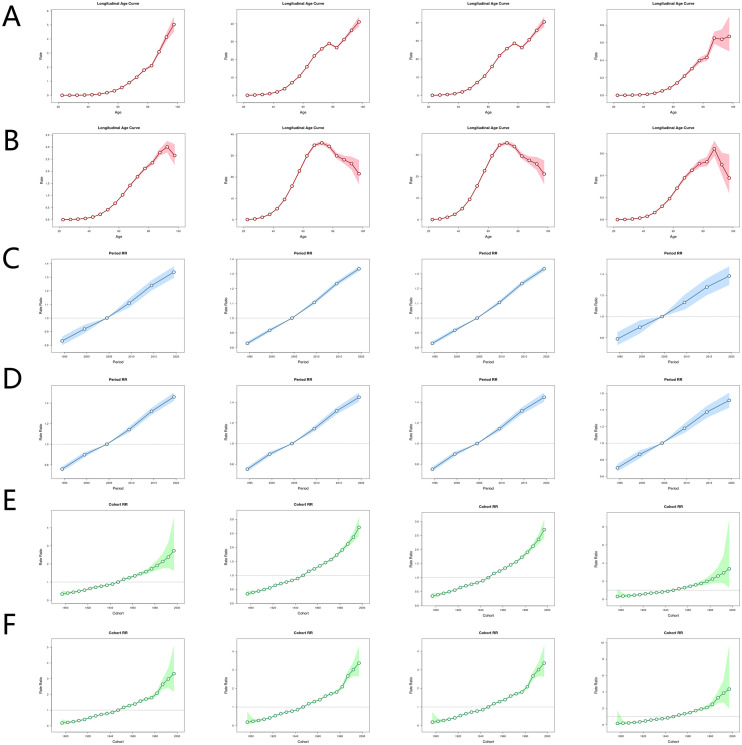
Age-period-cohort analysis results. Longitudinal Age Curve in deaths, DALYs, YLLs and YLDs for female **(A)** and male **(B)**. Period RR in deaths, DALYs, YLLs and YLDs for female **(C)** and male **(D)**. Period RR takes RR = 1 as the reference, representing no period effect. Cohort RR in deaths, DALYs, YLLs and YLDs for female **(E)** and male **(F)**. Cohort relative risk takes RR = 1 as the reference, representing no cohort effect. DALYs, disability-adjusted life years; ASMR, age-standardized mortality rate; ASDR, age-standardized disability-adjusted life years rate; YLLs, years lived with disability; YLDs, years of life lost. RR, relative risk. The shaded area represents the 95% uncertainty interval.

The Longitudinal Age Curve for Deaths shows low mortality risk in younger age groups with flat curves and narrow 95% UIs, indicating minimal high BMI impact on liver cancer mortality here and low estimation uncertainty. Mortality risk rises rapidly from 60 years (earlier in males), with accelerating growth as age advances; concurrently, 95% UIs widen, reflecting higher estimation uncertainty for older adults’ mortality risk. Female mortality risk differs, rising continuously with age, no decline even at 95 years. DALYs and YLLs follow similar trends: females decline around 80 years then rise, while males decline consistently after 75 years. YLDs differ slightly: female YLD growth slows at 80, rises sharply at 85, and shows flat decline (90 years) vs. upward trend (95 years); males follow this up to 80 years but YLDs drop rapidly after 85. Period RRs show consistent temporal patterns across all health outcomes and genders, with males having numerically higher RRs: 1995-2005 (RR< 1, weak high BMI-liver cancer association), 2005 (RR = 1), and post-2005 (RR stays above 1 and rises faster, indicating growing excess risk of liver cancer-related outcomes from high BMI). Cohort RRs have a simple upward trend, with males having numerically higher values: early cohorts (1900-1940, RR< 1), middle cohorts (1940-1960, RR = 1), and late cohorts (1960-2000, RR > 1). Concurrently, 95% UIs widen significantly (especially deaths/YLDs), reflecting higher estimation uncertainty; additionally, male Cohort RRs for 1985 birth cohorts rise abnormally fast, requiring special attention (**Figure 3**).

### 3.5 Result of joinpoint regression analysis

**Figure 4:**
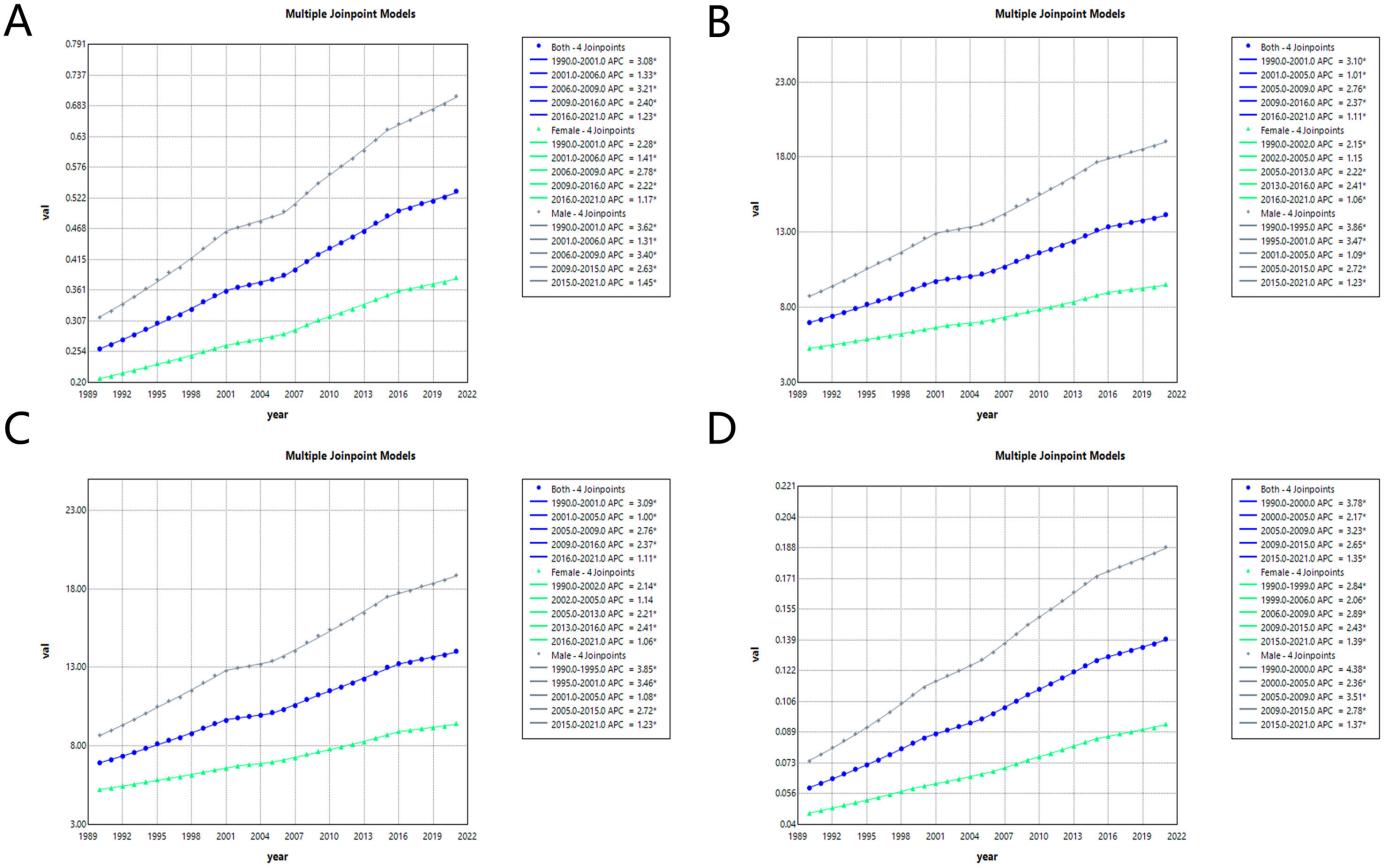
Joinpoint regression analysis of liver cancer attributable to high BMI burden temporal trends, 1990-2021. ASMR **(A)**, ASDR **(B)**, YLLs **(C)**, YLDs **(D)** for joinpoint regression analysis. DALYs, disability-adjusted life years; ASMR, age-standardized mortality rate; ASDR, age-standardized disability-adjusted life years rate; YLLs, years lived with disability; YLDs, years of life lost. Green represents females; gray represents males; purple represents both.

Regression analysis revealed rising ASMR and ASDR for liver cancer from 1990 to 2021, with the growth rate beginning to decline in 2016. YLLs and YLDs followed comparable patterns. Males experienced a higher disease burden than females, though trends were similar. Overall, ASMR increased, though growth slowed post-2016 (2016-2021APC: 1.23%; *p* < 0.01). ASDR also rose, with declines after 2016 (2016-2021APC: 1.11%; *p* < 0.01). YLLs climbed but dropped from 2016 (2016-2021APC: 1.11%; *p* < 0.01) onward. YLDs grew rapidly early (1990-2000APC: 3.78%; *p* < 0.01), then slowed, yet maintained an upward trend (2015-2021APC = 1.35%; *p* < 0.01) (**Figure 4**).

### 3.6 Analysis of regional differences and health inequalities

**Figure 5:**
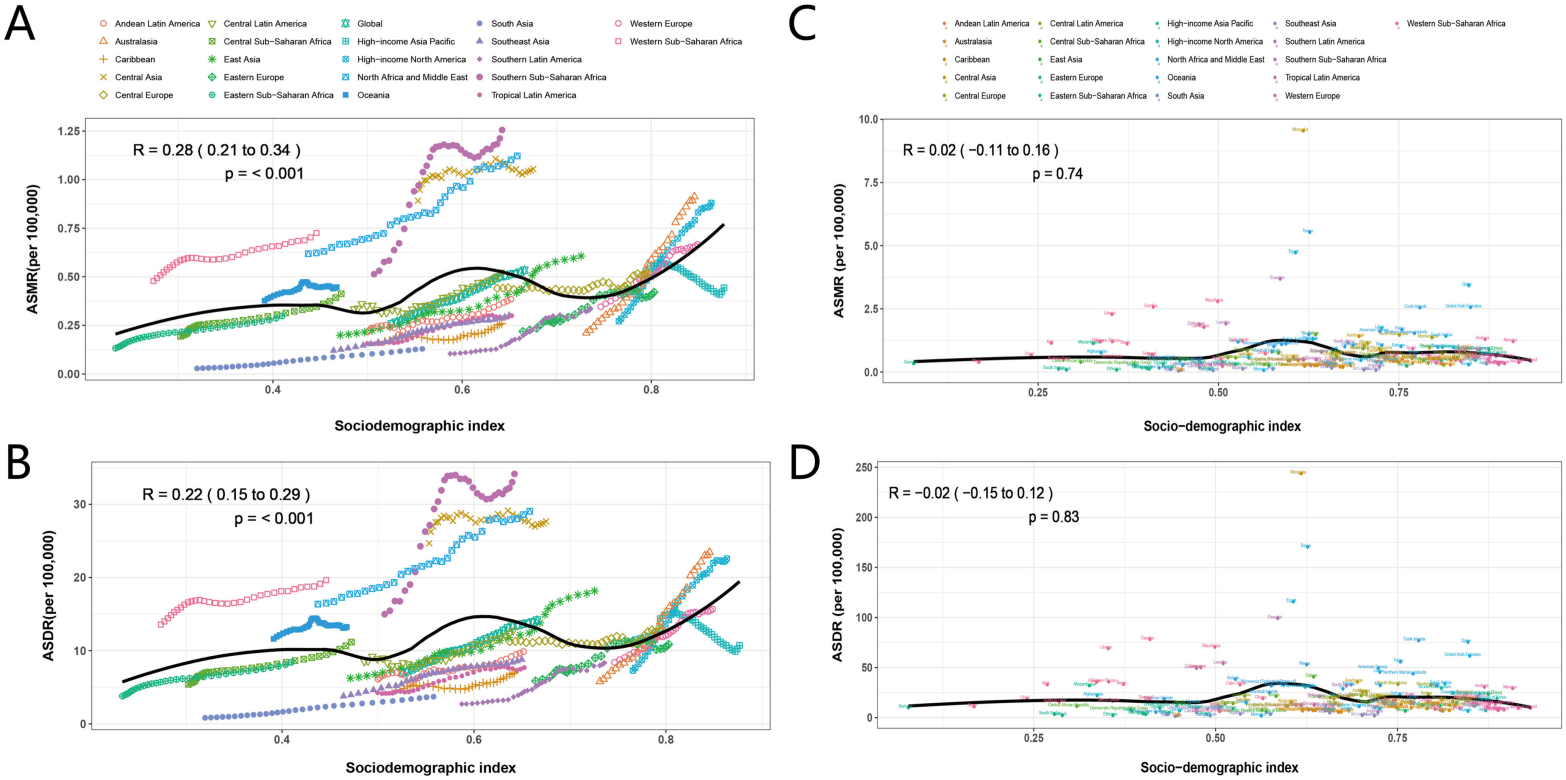
Correlation between ASMR, ASDR and SDI in liver cancer attributable to high BMI for 204 countries and 21 regions, 1990-2021. The above points show estimates for each country and region. The correlation between ASMR **(A)** or ASDR **(B)** and SDI in 21 GBD regions. The association between ASMR **(C)** or ASDR **(D)** and SDI in 204 countries. DALYs, disability-adjusted life years; ASMR, age-standardized mortality rate; ASDR, age-standardized disability-adjusted life years rate; SDI, sociodemographic index.

The relationship between SDI and ASMR (R = 0.55, 95% CI: 0.49-0.6, p<0.001) and ASDR (R = 0.56,95% CI: 0.5-0.61, *p* < 0.001) is nonlinear both globally and in the 21 GBD regions. At the top of the curve, Southern Sub-Saharan Africa, Central Asia, and North Africa and Middle East have higher ASMR and ASDR. Notably, High-income Asia Pacific has a low burden despite high SDI. Low SDI and Low-middle SDI regions have lower ASMR and ASDR. At the national level, the association remains significant in ASMR (R = 0.62,95% CI: 0.53-0.7, p< 0.001) and ASDR (R = 0.61,95% CI: 0.51-0.69, p< 0.001). The correlation between ASMR, ASDR, and SDI is similar. The disease burden in Mongolia is exceptionally high (**Figure 5**).

**Figure 6:**
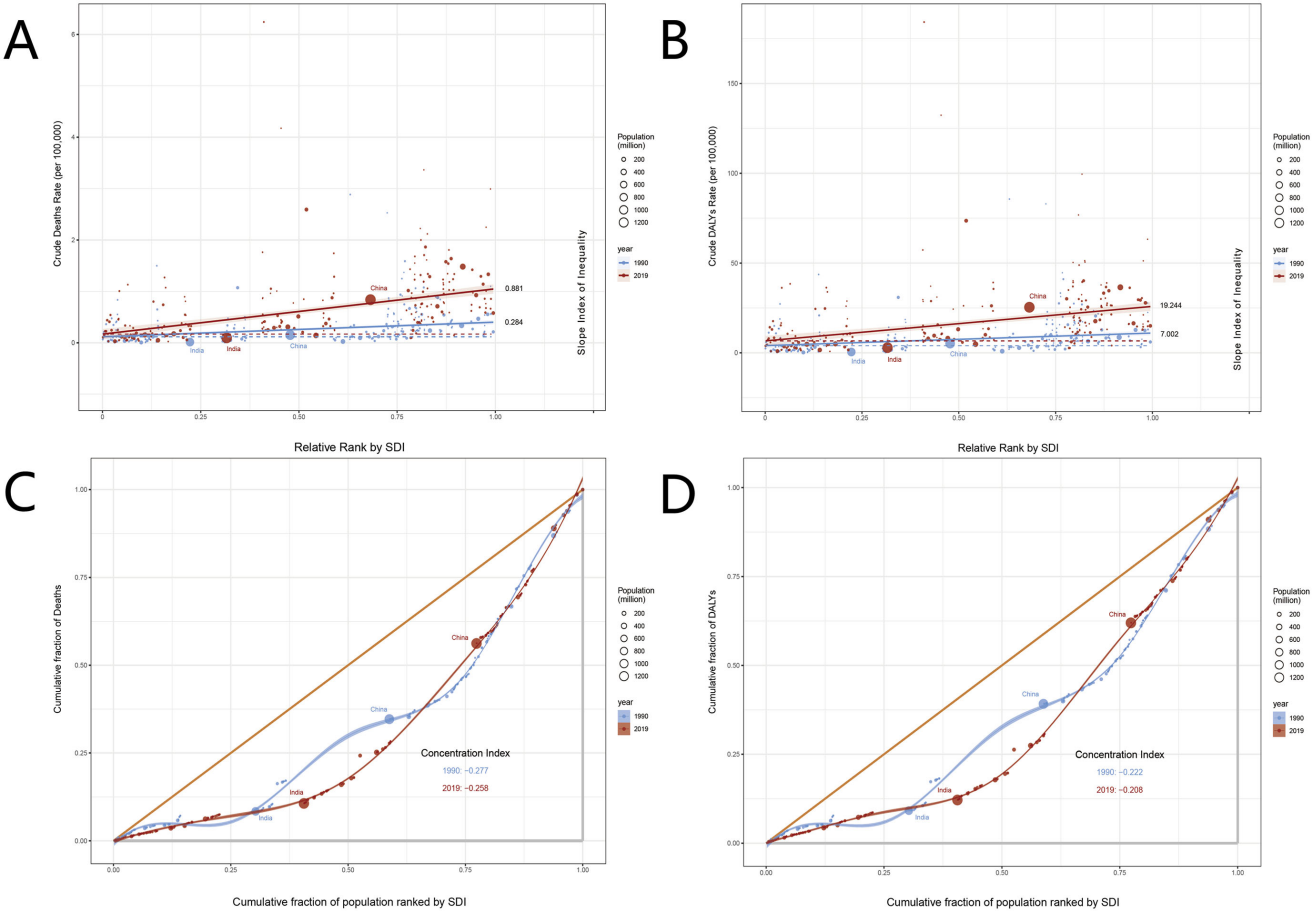
Analysis of deaths and DALYs inequality in liver cancer attributable to high BMI in 1990 and 2019. The 1990 and 2019 statistics are displayed in blue and red, respectively. Crude deaths **(A)** and DALYs **(B)** rate per 100,000 by SDI, 1990 and 2019, by relative rank. Cumulative fraction of deaths **(C)** and DALYs **(D)** rate by cumulative population percentage, ordered by SDI in 1990 and 2019. DALYs, disability-adjusted life years; SDI, sociodemographic index. Red represents 2019; blue represents 1990. The size of the circles represents population size (in millions). The shaded area represents the 95% uncertainty interval.

SII is positive, indicating that high socioeconomic status is associated with better health outcomes in the studied population. From 1990 to 2019, the crude mortality SII value decreased from 0.881 to 0.284, and the crude DALY rate decreased from 19.244 to 7.002, reflecting a reduction in global health inequality\. The concentration index is negative, suggesting that the disease burden is primarily concentrated in areas with lower SDI. From 1990 to 2019, the crude mortality concentration index increased from-0.277 to-0.258, and the crude DALY rate increased from-0.222 to-0.208, indicating a reduction in health inequality. China and India have large populations, so even small changes in proportions can have significant impacts on health equity issues. India has a lower SDI and a higher degree of health inequality. China has a relatively higher SDI, but there has been a significant increase in cumulative fraction of deaths and DALYs (**Figure 6**).

### 3.7 Frontier analysis and future prediction

**Figure 7:**
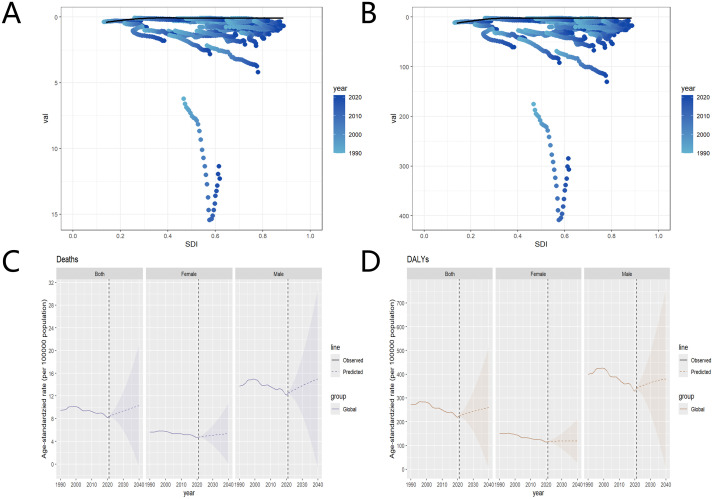
Trends in the association of the global burden of liver cancer attributable to high BMI with the SDI level, from 1990 to 2021. **(A)** ASMR; **(B)** ASDR. The black line represents the frontier, the lowest attainable disease burden threshold under the SDI level. The scatter points show actual data from different countries over the years. The color gradient indicates the years, with light blue representing 1990 and dark blue representing 2020. Future prediction by 2040 for liver cancer attributable to high BMI, both globally and by gender. **(C)** ASMR: **(D)** ASDR. Observed data are shown as solid lines showing past data. The predicted data are represented as dashed lines showing future trend predictions. The shaded area represents the range of uncertainty in the forecast. **Figure 5**. DALYs, disability-adjusted life years; ASMR, age-standardized mortality rate; ASDR, age-standardized disability-adjusted life years rate; SDI, sociodemographic index.

The frontier line is close to 0 and negatively correlated with the overall SDI. The scatter points are concentrated near the frontier line. From 1990 to 2021, the scatter points show an upward trend in ASMR and ASDR as the years progress. It is projected that by 2040, the ASMR and ASDR for liver cancer attributable to high BMI will continue to rise globally. The future forecast for males shows rapid growth, while the forecast for females shows a less pronounced change, though still an upward trend. The uncertainty range for the prediction is large, and it should be interpreted with caution (**Figure 7**).

## 4 Discussion

This study, based on GBD 2021 data, systematically evaluated the trend of global BMI-related liver cancer burden from 1990 to 2021. The results show that high BMI has become a significant driving factor for both the incidence and mortality of liver cancer. In 2021, the number of liver cancer deaths due to high BMI reached 46,200 globally, with a DALYs of 1,273,313, representing increases of approximately 2.1 times and 1.9 times, respectively, compared to 1990. This growth trend is closely linked to the global obesity epidemic and influenced by complex factors such as population aging (21), metabolic risk and regional socioeconomic inequality (22). Notably, the distribution of the disease burden exhibits significant heterogeneity, with —— male mortality and DALYs accounting for over 65%. East Asia, Southeast Asia, and West Africa have become high-risk areas for age-standardized mortality, while high-income countries, benefiting from better medical resources, bear relatively lighter burdens (23). These findings highlight the challenges faced globally.

Liver cancer linked to high BMI exhibits striking regional variations worldwide. Nations grappling with the highest disease burden often coincide with populations plagued by obesity—a condition fueled by diets rich in fats and sugars coupled with sedentary lifestyles. Over the past 30 years, the incidence and death rates of liver cancer in Mongolia have been steadily increasing, and in 2019, Mongolia had the highest standardized burden of liver cancer in the world (24). Recent findings from a 2022 Global Burden of Disease study on Mongolia (25) revealed a sharp uptick in liver cancer cases, strongly implicating suboptimal lifestyle and dietary choices. The WHO highlights four key behavioral risks—tobacco use, poor nutrition, physical inactivity, and excessive drinking—alongside four metabolic threats: hypertension, obesity, elevated blood glucose, and hypercholesterolemia, all of which heighten susceptibility to chronic diseases. A 2019 Mongolian survey revealed that 50% of the population is overweight, with 20% obese, significantly raising liver cancer risks. Most cancers in Mongolia are detected late, resulting in low survival rates—80% of liver cancer cases are identified at advanced, untreatable stages (26). Secondary prevention, including better access to diagnostic tools like endoscopy and CT scans, is essential. Similarly, sedentary habits and high-calorie diets in North America and Europe drive obesity-linked liver cancer risks. From 1990 to 2021, obesity-related liver cancer mortality surged in regions like the Caribbean, Persian Gulf, and Southeast Asia, highlighting a growing health burden. China’s fast-food sector has expanded 13% yearly since 2008, but public health infrastructure lags behind. Meanwhile, Northern Europe and Central Asia’s reduced EAPC likely stems from strict dietary regulations (e.g., sugar taxes, trans fat bans) and comprehensive health initiatives, such as Mexico’s NCD prevention programs targeting liver cancer (27). Key interventions involve food labeling, marketing restrictions for child-targeted products, and soda taxes.

This research explores liver cancer trends linked to high BMI, emphasizing notable gender and age disparities in public health. Findings show HCC mortality and disability are higher in men, peaking at 55–69 years versus 65–69 in female, suggesting obesity’s sex-specific pathways to liver cancer. Metabolic disorders from obesity alter sex hormone levels (28), with male prevalence tied to androgen-driven liver cell growth and inflammation. Estrogen modulates the ERα-36/AKT/Foxo3a pathway, lowering antioxidant enzyme production and increasing oxidative stress, promoting liver cancer cell death (29). The increased disease burden in older adults relates to reduced postmenopausal hormones (2) and immune aging. Mortality and DALYs peak and decline, possibly due to competition among age-related comorbidities like cardiovascular disease (30), especially in men. The drop in mortality among those aged 90-94+ may result from survivor bias. Persistent high burden in men and declining rates in female since 2010 suggest differing success in risk mitigation. While rising global obesity (11) explains initial increases, female’s decline may result from better early detection (31) and preventive care (32). Persistent obesity trends among men reveal gaps in current interventions targeting male-centric risk factors. A decade-long Chinese study of 500,000 adults (33) found that each extra 280g of weekly alcohol (about four daily drinks) increased liver cancer risk by 44% (HR 1.44, 95% CI 1.23-1.69), with pre-meal drinking showing notably higher risk (HR 1.32, 1.01-1.72).

Results from the Age-Period-Cohort Analysis show that in younger age groups, risk curves for all genders and indicators are flat, with narrow UIs. As age increases, the associated risk rises at an accelerating rate. High BMI exposure in younger populations is typically short, and MASLD requires long-term metabolic abnormality accumulation to manifest its risks (8). Younger individuals’ biological defenses, such as liver repair (34) and glucose regulation (35), mitigate obesity’s harms. A study of 31,505 MASLD patients (36) showed no significant difference in cirrhosis progression between young adults (18-<40 years) and older adults (≥60 years); however, younger adults had a significantly lower risk of liver-related events (LREs), including HCC. With age, immune aging and cellular energy depletion worsen the inflammatory and carcinogenic effects of obesity (37, 38). Aging impairs immune function, increasing chronic inflammatory responses and weakening immune surveillance. This promotes carcinogenesis, with HCC accounting for 28.4% of LREs in older groups (36). Obesity combined with diabetes raises the risk of cirrhosis and HCC with advancing age (36). The finding that female mortality risk does not decline is consistent with earlier results. DALYs, influenced by mortality and age, show synchronized trends, with males experiencing rapid declines in YLLs after 85 years, unlike females, where YLLs initially rise before stabilizing. Period effects show a weak association between high BMI and liver cancer from 1995 to 2005, but after 2005, RR accelerates, reflecting increased obesity prevalence. By 2020, obesity rates had surged, and high BMI became a major risk factor for liver cancer. Differences in male and female Period RRs are due to males’ greater metabolic sensitivity to high BMI and higher alcohol consumption, which synergistically increase liver cancer risk (39). Cohort effects reveal that high BMI exposure has increased across generations, particularly in younger cohorts, due to changing dietary and lifestyle habits. This trend explains the rapid rise in risk for males born around 1985, influenced by global fast-food expansion. Widening CIs in late cohorts reflect uncertainty due to insufficient data and varied exposure patterns.

The statistical analysis of liver cancer trends from 1990 to 2021 reveals a clear correlation between rising BMI levels and increased hepatocellular carcinoma incidence, mirroring worldwide surges in obesity, metabolic disorders, and demographic aging-primary factors in NAFLD’s progression to liver cancer. Interestingly, the growth rate began decelerating post-2016, suggesting that although public health measures took time to implement, their effectiveness became apparent through earlier diagnosis and better treatment outcomes. Post-2015 research indicates metformin may lower HCC risk in diabetic NAFLD patients, potentially slowing progression in high-risk groups. Despite increased survival, DALYs continue rising, with YLDs growing despite declining YLLs. Systemic treatments like immune checkpoint inhibitors extend survival but may worsen disability due to side effects and recurrence, highlighting the need to integrate palliative care throughout HCC management, not just at end-stage.

Socioeconomic development and high BMI-related liver cancer burden share a complex link, with health disparities slightly improving. The analysis of health inequalities presented here is based on the Global Burden of Disease Study 2019 data, and thus the results should be interpreted within the context of this time frame. Nations like the Mongolia face heavier burdens than Japan, South Korea, and Singapore. NAFLD, prevalent in obesity, affects 10% of developed nations, with 2-3% of NASH cases progressing to cirrhosis yearly and 10-20% to HCC (40). The Japanese Society of Obesity (JASSO) considers a BMI of 22 kg/m² as the optimal healthy weight, classifying obesity at ≥25 kg/m². Japanese men average a BMI of 24 kg/m², while female maintain 22 kg/m². Japan’s obesity-related mortality rate (3.2‰) is 60% lower than the U.S. (8.1‰), highlighting the impact of policies and cultural habits like moderate alcohol consumption and fish-based diets in reducing obesity-linked diseases (41). Although East Asia’s rising SDI has minimally increased health burdens, traditional diets and active lifestyles may mitigate metabolic risks—though this protective effect is declining with younger generations. In medium SDI regions (e.g., Southeast Latin America, Central and Eastern Europe), adapting healthcare infrastructure to the transition from infectious to chronic diseases is vital. Early screening can reduce obesity-linked liver cancer risks during initial SDI growth. High SDI, high-burden nations (e.g., the Mongolia) must optimize healthcare-policy coordination to address resource disparities and high BMI-related liver cancer. High SDI, low-burden countries (e.g., Japan, South Korea, Singapore) should prioritize cultural health preservation. Low/medium-low SDI areas must focus on obesity prevention to avert future disease burdens. For severe obesity (≥100 kg), weight-loss surgery is a safe, effective intervention, significantly lowering HCC risk (42).

The frontier line represents the lowest achievable disease burden at a specific SDI level, with its low level indicating substantial effectiveness of current global prevention and control strategies, and this effectiveness is reflected across countries at different SDI levels. The low level of the frontier line means that, under current medical technologies and public health resource allocation, countries across all SDI levels can suppress the burden of high BMI-related liver cancer to a lower threshold through scientific prevention and control. For example, Japan has implemented targeted measures for key factors of high BMI-related liver cancer (obesity, NAFLD/NASH, diabetes, genetic susceptibility), effectively controlling the disease burden (42). The Japan Society for the Study of Obesity (JASSO) defines a BMI of 22 kg/m² as an appropriate weight and BMI ≥25 kg/m² as overweight, aligning with the metabolic characteristics of the Japanese population and providing a clear basis for weight management. Japan includes NAFLD patients in the high-risk liver cancer screening system, using ultrasound and fibrosis markers for early diagnosis. In 2021, the early diagnosis rate of NASH-related liver cancer reached 58%. At the same time, diabetes is co-managed to block comorbidity risks, and high-risk populations with the PNPLA3 GG genotype are screened. China reduces the disease burden through the hepatitis B vaccine and screening in high-incidence liver cancer areas in rural regions, approaching the frontier line at a low level. The projected continued rise in the global burden of liver cancer attributable to high BMI by 2040 warrants attention. A study using the GLOBOCAN database also predicts that by 2040, the incidence of primary liver cancer will continue to increase (43). The large uncertainty range suggests that the future trend of the burden is not fixed and depends on key variables such as the strength of obesity prevention and control measures and advances in medical technologies. The large range of the prediction interval stems from the multiple uncertainty assumptions relied upon by the model. The prevalence of obesity is the result of individual behavior changes, population structure transitions, and macro-environmental drivers, with changes in individual behaviors and states across the entire population being central (44). These factors carry a high degree of uncertainty. At the same time, advances in liver cancer diagnostic and treatment technologies, such as new early screening methods and the development of breakthrough drugs, may exceed model expectations, significantly altering the disease incidence and mortality trajectory (45). Therefore, this prediction should be seen as a possible projection based on current data and trends. Future projections should be dynamically adjusted based on real-time monitoring data, highlighting the importance of long-term disease burden monitoring and assessment for precise public health decision-making.

Our study systematically evaluated the overall trends of liver cancer attributable to high BMI globally from 1990 to 2021. The integration of comprehensive data and multi-method analysis enhanced the robustness of the findings, providing actionable insights for prevention strategies in specific regions. However, some limitations must be acknowledged. First, in resource-poor areas, cancer registration coverage is inadequate, forcing us to rely on model estimates that may understate the actual burden due to insufficient diagnosis or incomplete reporting. Second, diagnostic heterogeneity, such as changes in screening practices (like incidental detection versus symptomatic testing) and evolving ICD coding standards, can distort time trends, especially in high-income regions with advanced imaging technology. Third, genetic predispositions, co-infections such as HBV/HCV, or residual mixtures of environmental carcinogens like aflatoxins may affect the association between BMI and liver cancer. Fourth, the age-period-cohort model assumes a linear relationship for cohort effects, which might oversimplify the nonlinear interaction between long-term obesity exposure and liver cancer incidence. Finally, although SDI is closely related to liver cancer attributable to high BMI burden, mortality competition in low SDI areas can result in a lower disease burden.

## 5 Conclusion

The escalating burden of liver cancer attributable to high BMI underscores the critical intersection of obesity, aging, and socioeconomic inequality. Prioritizing early metabolic screening, gender-specific prevention strategies, and equitable healthcare access is essential to curb this trend. Future projections show that the burden of high BMI-related liver cancer will continue to rise until 2040, with males facing a faster increase than females. Future efforts must integrate longitudinal biomarker studies and policy reforms to address emerging risks.

## Data Availability

The datasets presented in this study can be found in online repositories. The names of the repository/repositories and accession number(s) can be found in the article/supplementary material.
